# Antibacterial and Inhibitory Activity of Nora and Mepa Efflux Pumps of Estragole Complexed to β-Cyclodextrin (ES/β-CD) In Vitro Against *Staphylococcus aureus* Bacteria, Molecular Docking and MPO-Based Pharmacokinetics Prediction

**DOI:** 10.3390/pharmaceutics16111469

**Published:** 2024-11-18

**Authors:** Roger Henrique Sousa da Costa, Renata Torres Pessoa, Eduardo dos Santos Silva, Isaac Moura Araujo, Sheila Alves Gonçalves, Janaína Esmeraldo Rocha, Francisco Nascimento Pereira Junior, Naiara Cipriano Oliveira, Victor Moreira de Oliveira, Matheus Nunes da Rocha, Emmanuel Silva Marinho, Natália Kelly Gomes de Carvalho, José Galberto Martins da Costa, Hélcio Silva dos Santos, Irwin Rose Alencar de Menezes

**Affiliations:** 1 Veterinary Medicine Course, Maurício de Nassau University Center, Juazeiro do Norte 63010-475, CE, Brazil; rogerhenrique8@hotmail.com; 2 Laboratory of Pharmacology and Molecular Chemistry (LFQM), Department of Biological Chemistry, Regional University of Cariri, Rua Coronel Antônio Luis 1161, Pimenta, Crato 63105-000, CE, Brazil; renata.pessoa@urca.br (R.T.P.); eduardodos.santos@urca.br (E.d.S.S.); 3 Laboratory of Microbiology and Molecular Biology, Department of Biological Chemistry, Regional University of Cariri, Rua Coronel Antônio Luis 1161, Crato 63105-000, CE, Brazil; isaac.moura@urca.br (I.M.A.); sheila.alves@urca.br (S.A.G.); janainaesmeraldo@gmail.com (J.E.R.); 4 Center for Agricultural and Biodiversity Sciences, Federal University of Cariri, Crato 63130-025, CE, Brazil; francisco.pereira@ufca.edu.br; 5 Department of Physics, Regional University of Cariri, Juazeiro do Norte 63041-145, CE, Brazil; naiara.cipriano@urca.br; 6 Program in Natural Sciences, State University of Ceará, Fortaleza 60714-903, CE, Brazil; vitor.moreira@aluno.uece.br (V.M.d.O.); nunes.rocha@aluno.uece.br (M.N.d.R.); emmanuel.marinho@uece.br (E.S.M.); 7 Laboratory of Research and Natural Product (LPPN), Department of Biological Chemistry, Regional University of Cariri, Rua Coronel Antônio Luis 1161, Pimenta, Crato 63105-000, CE, Brazil; nataliakellygc@gmail.com (N.K.G.d.C.); galberto.martins@urca.br (J.G.M.d.C.); 8 Center for Exact Sciences and Technology, Vale do Acaraú University, Sobral 62040-370, CE, Brazil

**Keywords:** efflux pump, terpenes, bacterial resistance, estragole

## Abstract

**Background/Objectives:** The work investigates the effect of the estragole complex encapsulated in beta-cyclodextrin (ES/β-CD) in modulating bacterial resistance, specifically in *Staphylococcus aureus* strains expressing NorA and MepA efflux pumps. Efflux pumps are mechanisms that bacteria use to resist antibiotics by expelling them from the cell. Methodology: Several compounds and antibiotics, such as ciprofloxacin and norfloxacin, were used to evaluate the antimicrobial activity and the ability of the ES/β-CD complex to reverse resistance. **Methods:** The study included scanning electron microscopy assays, minimum inhibitory concentration (MIC) determination, and efflux pump inhibition tests. **Results:** The ES/β-CD complex did not show significant direct antibacterial activity. However, it modulated the action of norfloxacin, decreasing the MIC when combined with this antibiotic in the 1199B (NorA) strain. These results suggest a potential for synergy but not a direct inhibition of efflux pumps. Conclusion: ES/β-CD can potentiate the efficacy of some antibiotics but does not directly act as an efflux pump inhibitor; it is more of an antibiotic potentiator than a direct solution to bacterial resistance. The molecular docking simulation data suggest its high affinity for forming the ES/β-CD complex. The pharmacokinetic predictions based on MPO suggest that the compound has moderate lipophilicity, highly effective cellular permeability, and low incidence of organic toxicity, pointing to a promising pharmacological principle with controlled daily oral dosing. **Conclusions:** These results indicate this complex’s possible and relevant association as an adjuvant in antibiotic therapy to reduce multidrug-resistant bacteria; however, new in vivo assays are necessary to confirm this effect.

## 1. Introduction

*Staphylococcus aureus* (SA) is a Gram-positive, facultatively anaerobic bacteria that exhibits respiratory metabolism but can grow by fermentation. It causes major health problems worldwide due to its ability to cause skin and soft tissue infections, which can evolve into more severe and invasive infections [1].

Bacterial resistance is responsible for significant clinical and economic impacts, increasing morbidity and mortality rates. *S. aureus*, in turn, is among the Gram-positive bacteria that present several specific strategies to promote antibiotic resistance. The excessive use of drugs that target these types of bacteria leads to the development of resistance mechanisms. These can include genetic mutations that alter the antibiotic’s binding site or the inactivation of the drug through hydrolysis, which renders the antibiotic ineffective [2].

Integral membrane transporters are drug efflux pumps and are one of the mechanisms of bacterial resistance to antibiotics and biocides [3]. When bacteria are exposed to antibiotics and biocides, various reactions are triggered that lead to increased expression of a group of membrane transport proteins responsible for efflux, commonly known as the multidrug efflux system. This system provides several efflux pathways that function cooperatively and are responsible for the multidrug resistance phenotype [4,5]. Efflux pumps can be genetically encoded in the bacterial central chromosome, as is the case for the efflux pumps NorA (MFS family) and MepA (MATE family), or encoded on the plasmid, as in the pumps MsrA (ABC superfamily), QacA and QacB (MFS), and QacC (SMR family) [6].

In this context, researchers have begun searching for new therapies of natural origin as alternatives to combat the pathologies caused by these microorganisms and have proposed new strategies to reverse bacterial resistance [7,8]. Among these compounds found in plants are terpenes produced by plants, which provide protection against biotic and abiotic stresses. These compounds are widely studied due to their great structural variety, including linear hydrocarbons or carbocyclic skeletons [9]. Many terpenoids, including estragole [10], have been reported to have several therapeutic properties, such as antimicrobial effects [11] and modulation of bacterial resistance by efflux pump inhibition [12,13], anti-inflammatory [14,15], and immunomodulatory activity [16]; thus, these compounds are of great interest in the medical field. However, this class has low solubility in water and is prone to volatilization, making its interaction and formulation in therapeutic systems difficult [17]. To solve this problem, encapsulation with beta cyclodextrin becomes a significant alternative, considering that this nanoencapsulation can increase stability and bioavailability and facilitate these compounds’ solubility [18,19,20,21].

In light of the above, this study aims to evaluate the antibacterial activity and capacity of estragole complexed in beta-cyclodextrin (ES/β-CD) to reverse the efflux pump resistance mechanism of *S. aureus* strains carrying the NorA and MepA pumps. In addition, molecular docking and MPO-based pharmacokinetics prediction were carried out.

## 2. Materials and Methods

The substances used, such as estragole, chlorpromazine (CPZ), carbonyl cyanide-m-chlorophenylhydrazone (CCCP), and ethidium bromide, were obtained from Sigma Aldrich Co., Ltd. (St. Louis, MO, USA). Antibiotics (Ciprofloxacin and Norfloxacin), and estragole were dissolved in dimethyl sulfoxide (DMSO) and then in pure water. CPZ and ethidium bromide solutions were dissolved in sterile distilled water and protected from light. CCCP was dissolved in a methanol/water solution in a 1:1 ratio. The final concentration of all compounds was 1024 µg/mL. The culture media used in the study were brain heart infusion BHI, and agar BHI obtained from Acumedia Manufacturers Inc. (Lansing, MI, USA) prepared according to the manufacturer, at a concentration of 10%.

### 2.1. Estragole Inclusion Complex in β-Cyclodextrin (ES/β-CD)

The ES/β-CD inclusion complex was prepared according to the procedure described by da Costa et al. (2022) [22]. In turn, the complex was stored in amber glass for use in the assays.

#### 2.1.1. Scanning Electron Microscope Images of ES/β-CD

To capture images of the morphology of β-CD and ES/β-CD, a SU3500 microscope (Hitachi, Tokyo, Japan) was used. Samples were prepared by spraying them onto carbon tape then mounting them on an aluminum stub. No additional coating was applied. SEM images were acquired in high vacuum mode at a working distance of 6.4 mm using a secondary electron detector and an electron acceleration voltage of 5 kV.

#### 2.1.2. Chemical Analysis by GC-FID

The sample and standard were analyzed using a Shimadzu GC-2010 Plus equipped with a flame ionization detector (FID) supplied by Shimadzu Scientific Instruments Inc. (Columbia, MD, USA) with a SH-Rtx-5 fused silica capillary column (30 m × 0.25 mm I.D.; 0.25 m film thickness) and the following temperature program: 80–180 °C at 4 °C/min, then to 246 °C at 6.6 °C/min, ending with 10 min at 280 °C at 3.4 °C/min, for a total analysis time of 30 min. Helium gas was used as the carrier gas, with a flow rate of 1.5 mL/min, split mode (1:15), and the injection port was set to 220 °C. Injection volume: 1 µL of sample and standard solution prepared with dichloromethane. The detector temperature was set to 300 °C. The linear retention index was obtained by injecting a mixture of linear C8-C40 hydrocarbons under the same conditions as the samples. The identity of the compounds was confirmed by comparing their retention indices and mass spectra with those taken from the literature [23].

Quantification was performed by integrating the peaks using the external standard method. The quantifications of the compounds were based on analytical curves of the reference standards. The limit of detection (LOD) and the limit of quantification (LOQ) were calculated based on the standard deviation of the responses and the slope using three independent analytical curves. The LOD and LOQ were calculated as 3.3 and 10 σ/S, respectively, where σ is the standard deviation of the response and S is the slope of the calibration curve. The estragole standard was diluted in 99.9% UV/HPLC-grade dichloromethane to create a stock solution at a concentration of 5000 ppm. This stock solution was further diluted to achieve concentrations ranging from 100 to 1000 ppm. For quantitative analysis, a complex formed by estragole and β-cyclodextrin was prepared at a concentration of 50 ppm.

### 2.2. Bacterial Strains

The bacterial strains of *S. aureus* used were SA-1199B (NorA-overexpressing) and SA-K2068 (MepA pump-expressing). The strains were provided by Prof. S. Gibbons (University of London) and maintained on blood agar (Laboratórios Difco Ltd., São Paulo, Brazil). Before the experiments, they were cultured for 24 h at 37 °C on solid brain heart infusion (BHI) agar medium (BHI, Acumedia Manufacturers Inc.).

### 2.3. Determination of Minimum Inhibitory Concentration (MIC)

The minimum inhibitory concentration (MIC) was determined using a microdilution assay according to the standards set by the Clinical and Laboratory Standards Institute (CLSI (2019)) with modifications [24]. The compounds were initially diluted in DMSO or saline to create stock solutions with a final concentration of 1024 μg/mL. These stock solutions were then subjected to serial dilution at a 1:1 ratio using brain heart infusion (BHI) in Eppendorf tubes, resulting in concentrations ranging from 0.5 to 512 µg/mL. For each test, a bacterial inoculum was prepared using 100 μL of a solution standardized to 0.5 on the McFarland scale, suspended in saline. The resulting mixture of the inoculum and the assayed compounds was then transferred to 96-well microtiter plates and incubated at 37 °C for 24 h. Bacterial growth was measured using resazurin. A control was created using a vehicle DMSO solution at 2%. The minimum inhibitory concentration (MIC) is the lowest concentration at which no bacterial growth is observed. Each assay was conducted in triplicate, and the results were reported as the average of the replicates.

### 2.4. Evaluation of NorA and MepA Efflux Pump Inhibition

Efflux Pump inhibition was tested using a Sub-inhibitory Concentration (MIC/8) of ES/β-CD and using the Standard Efflux Pump Inhibitors (SPI), CCCP and CPZ to verify the responses on the pumps tested, according to the methodology proposed by Oliveira-Tintino et al., 2018 [25]. All tests were performed in triplicate.

### 2.5. Molecular Docking Study

Molecular docking simulations were used to estimate the prediction of the interaction between beta-cyclodextrin (β-CD) and estragole (ES). The structure was configured to act as a target molecule to perform the simulations with cyclodextrin, while ES acted as a ligand in the interaction relationship. The structure of β-CD was acquired from the virtual repository of the Protein Data Bank (PDB) using the identification code IDpdb: 3M3R. For the structure “Crystal structure of the M113F alpha-hemolysin mutant complexed with beta-cyclodextrin” [26], the crystalline structure has co-crystallized β-CD at its center. Using the three-dimensional coordinates of the biological receptor, the system was inserted into the chimera software [27] to isolate only β-CD, which allowed the molecule to act as a target in molecular docking simulations.

Among the preparation steps, the two-dimensional coordinates of the ES were obtained from Pubchem through the identification code CID: 8815, and the initial information and coordinates of the ligand were entered into Avogadro software [28,29] to perform the electronic and structural optimization with the aim of reaching the lowest energy state within the metrics of the MMFF94 force field [30]. After the preparation of the β-CD (target molecule) and the ES (ligand), both structures were entered into the AutoDockTools software [31] to be standardized. Because β-CD was considered to be the target molecule, it was necessary to estimate its coordinates for action in the molecular docking simulations. In this way, the grid box axes were adjusted, presenting the following values: x = 33,948, y = 33,317 and z = 23,355, with the following sizes: 56 Å, 42 Å and 46 Å, related to the coordinates x, y and z, respectively. These calculations involved the entire β-CD structure.

The molecular docking simulations between β-CD and ES were performed with the support of the Auto Dock Vina software [32], where for the formed complex (target molecule/ligand), 50 independent simulations were performed with 20 possibilities for distinct interactions between the target and the ligand. This way, the configuration of greatest affinity for this relationship was presented, and then the best pose resulting from the most favorable simulation was formed. 

### 2.6. MPO-Based Pharmacokinetics Prediction

The pharmacokinetic properties of estragole were estimated using Pfizer, Inc.’s (New York, NY, USA) multiparameter optimization (MPO) drug-likeness scoring system, utilizing the MarvinSketch^®^ academic license program version 24.3.0, Chemaxon© (https://chemaxon.com/marvin), as shown in Equation (1):

d = ∑_(i = 1)^n w_k T_k (x^0^_k) (1)

where the desirability order (d) is given by the weighting factor (w) assigned to each physicochemical property (k) calculated in relation to the statistical thresholds (T(x)) formed by intrinsic lipophilicity (logP) ≤ 3, buffer lipophilicity (logD) ≤ 2, molecular weight (MW) ≤ 360 g/mol, Topological Polar Surface Area (TPSA) 40–90 Å^2^, H-bond donors (HBD) count ≤ 1, and the most basic pKa ≤ 8 (n = 6); resulting in a score that ranges from 0 to 6 according to pharmacokinetic viability [33].

The topological analyses were related to the descriptors of the Parallel Artificial Membrane Permeability Assay (PAMPA) predicted using online tools ADMETlab 3.0 (https://admetlab3.scbdd.com/), Deep-PK (https://biosig.lab.uq.edu.au/deeppk/), PreADMET (https://preadmet.qsarhub.com/), and ADMET-AI (https://admet.ai.greenstonebio.com/), which include effective cellular permeability (Papp, A→B) in colorectal adenocarcinoma (Caco-2) and Madin–Darby Canine Kidney (MDCK) cell lines, the hepatic clearance rate of the intrinsic molecular fraction (CL int,u), passive efflux by P-glycoprotein (P-gp), and volume of distribution (VD).

The prediction of metabolism sites was carried out using structural tracking models based on machine learning. The XenoSite (https://xenosite.org/) and StopTox (https://stoptox.mml.unc.edu/) servers were used. The results, which were generated from 2D susceptibility charts, were associated with metabolism descriptors dependent on isoforms CYP450 2C9, 2D6, and 3A4, predicted by the ADMETlab 3.0, Deep-PK, and PreADMET servers. Additionally, the prediction of a lethal dose (LD50) in rats, considering different administration routes, was conducted using the GUSAR Acute Rat Toxicity tool (http://www.way2drug.com/GUSAR/acutoxpredict.html).

### 2.7. Statistical Analysis

The data were analyzed through a two-way ANOVA test. The geometric mean of the triplicates were used as the central data and the standard deviation, and the statistical program GraphPad Prisma 7.0 was used. Then, a post hoc Bonferroni test was performed (*p* < 0.05 and *p* < 0.0001 are considered significant and *p* > 0.05 is not significant). The results of the molecular docking indicated that the relationship of the ES-β-CD complex could trigger a favorable process for system formation, and this was directly supported by evaluation parameters such as the interaction energy value, changes in RMSD, and the formation of three unconventional hydrogen bonds.

## 3. Results

### 3.1. Characterization of the Inclusion Complex

#### 3.1.1. Scanning Electron Microscope Images of ES/β-CD

**Figure 1:**
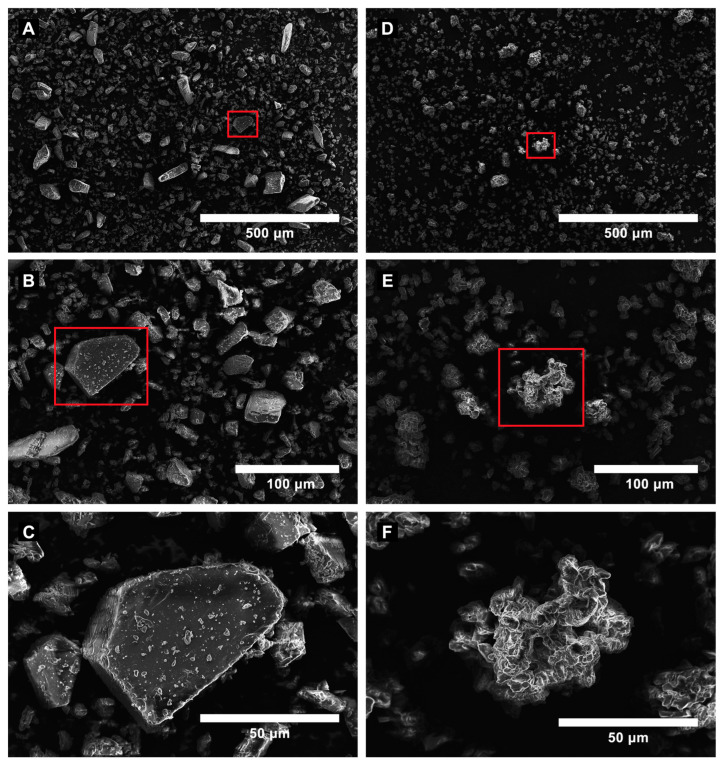
SEM images of pure β-cyclodextrin (β-CD) (**A**–**C**) and β-cyclodextrin associated with estragole (ES/β-CD) (**D**–**F**) are presented. To provide further details of the surface morphology before and after estragole association, higher-magnification images of the regions highlighted in red ((**A**,**B**) for β-CD, and (**C**,**D**) for ES/β-CD) are shown in (**C**,**F**), respectively.

Scanning electron microscopy (SEM) was used to examine the surface morphology of pure β-cyclodextrin (β-CD) and ES/β-CD. Pure β-CD exhibited irregularly shaped crystals of varying sizes (Figure 1A–C). In contrast, ES/β-CD showed amorphous structures (Figure 1D–F) resulting from the complexation, further supporting the other data reported in the literature [34,35] and corroborating the FID analysis.

Analysis of Figure 1A, performed using the granulometry tool of ImageJ (Version 1.54k), showed that particles appeared with a uniform distribution and calculated area of 4.27. At the same time, Figure 1D also presented a uniform distribution, but with particles of the smallest size and area calculated at 3.13. The particles depicted in Figure 1C are geometrically well defined and angular, featuring smooth surfaces and well-marked edges, which are typical of crystalline structures. In contrast, Figure 1F illustrates that the particles exhibit a more irregular shape, featuring rough edges and a porous structure. These variations highlight the structural and surface alterations, suggesting that the material has lost its crystalline form. This implies the formation of an inclusion complex, which generally leads to a more amorphous or disordered morphology that occurs when β-CD forms such complexes.

#### 3.1.2. Characterization of the Complex by GC/FID

**Figure 2:**
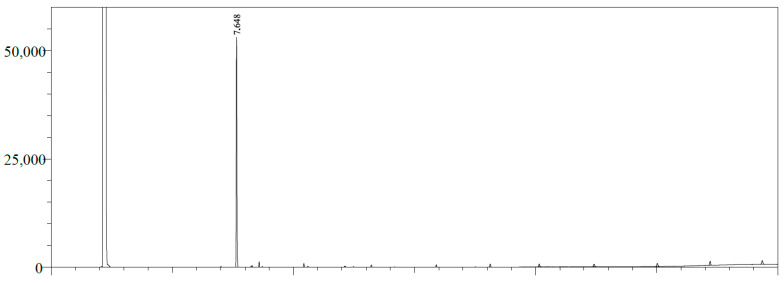
GC/FID profile of the estragole pattern (RT: 7.648 min).

**Figure 3:**
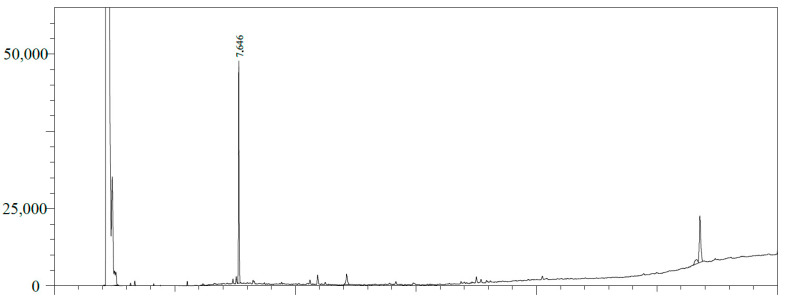
GC/FID profile of the β-cyclodextrin complex in estragole. Estragole (RT: 7.646 min).

The estragole content in the inclusion complex was analyzed using gas chromatography with a flame ionization detector (GC/FID), as shown in Figure 2 and Figure 3. The quantification was performed by integrating the peaks using the external standard method using the calibration curve for estragole with equation y = 953.98x + 1744.2 (r^2^ = 0.9976). The limit of detection (LOD = 0.05198 µg/mL) and limit of quantification (LOQ = 0.173266 µg/mL) were calculated based on the standard deviation of the responses and the slope using three independent analytical curves. Based on this calibration curve, the quantification of estragole was calculated at 12.726 ppm, corresponding to 25.45% of encapsulation in β-cyclodextrin, corroborating the literature data [36,37].

### 3.2. Minimum Inhibitory Concentration

The estragole complex with β-cyclodextrin obtained a minimum inhibitory concentration ≥ 1024 µg/mL. This was considered to be a clinically irrelevant concentration, since the amount required to obtain an effective plasma concentration would be very high.

### 3.3. Activity of Estragole Complexed to β-CD (ES/β-CD) Combined with Antibiotic and Ethidium Bromide

**Figure 4:**
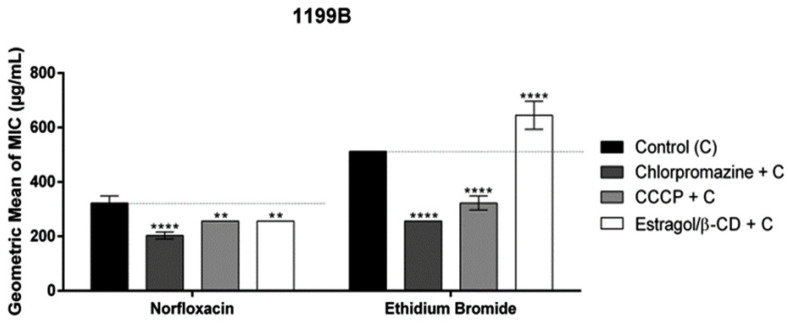
Evaluation of efflux pump inhibition of the products when associated with norfloxacin and ethidium bromide against *S. aureus* strain-overexpressing NorA efflux pumps. Each result is expressed as geometric mean ± geometric standard deviation (SD) of three simultaneous experiments. Statistical significance was determined by one-way ANOVA and Bonferroni’s post hoc test. The control contains only the vehicle solution of dimethyl sulfoxide (DMSO). (** *p* < 0.01, **** *p* < 0.0001 when compared to the negative control group).

For strain 1199B, when associated with norfloxacin, a combination of ES/β-CD and the antibiotic resulted in a decreased inhibitory concentration compared to that of the antibiotic alone, as observed for the CPZ and CCCP pump inhibitors. However, when associated with ethidium bromide, the complex increased its MIC. These results may suggest that the complex may exert a potentiating activity on the antibiotic, but not through the efflux pump (Figure 4).

**Figure 5:**
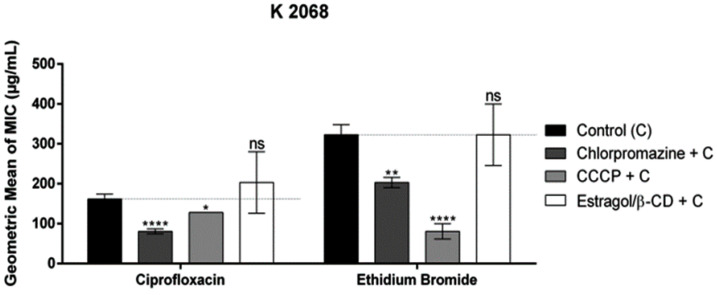
Evaluation of efflux pump inhibition of the products when associated with ciprofloxacin and ethidium bromide against *S. aureus* strain-overexpressing MepA efflux pumps. Each result is expressed as geometric mean ± geometric standard deviation (SD) of three simultaneous experiments. Statistical significance was determined by one-way ANOVA and Bonferroni’s post hoc test (ns—no significance; * *p* < 0.05; ** *p* < 0.01, **** *p* < 0.0001 when compared to the negative control group).

However, for the K2068 strain (Figure 5), the association of the complex with ciprofloxacin caused an increase in the MIC, although a statistically irrelevant one, compared to ciprofloxacin alone. When associated with ethidium bromide, the complex caused no difference in the control.

In previous findings, we demonstrated the effect of estragole on inhibiting efflux pumps [10,11]. However, the new data indicate that ES/β-CD does not directly inhibit efflux pumps like NorA and MepA, as shown in the ethidium bromide assay. The observed reduction in MIC for norfloxacin might be explained by an indirect mechanism, possibly due to alterations in the overall cellular environment or changes in membrane permeability caused by cyclodextrin, which could indirectly affect the efficacy of these pumps. In our in vitro assays, transferring estragole from β-CD to a hydrophilic medium is difficult due to the stabilization of estragole within the cyclodextrin cavity. These results are consistent with findings from other studies conducted by our group [11,38].

### 3.4. Molecular Docking Study

**Figure 6:**
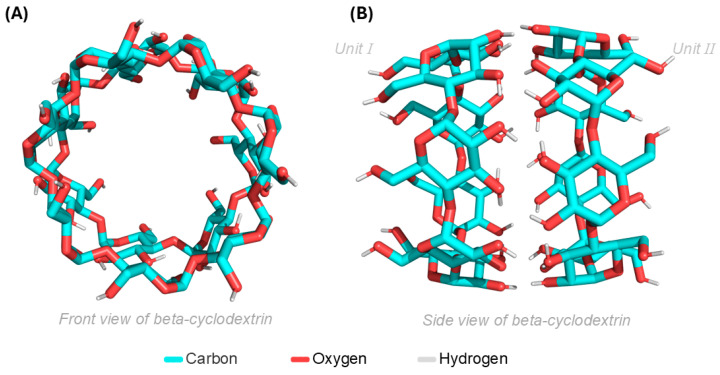
(**A**) Frontal view of β-CD in two units and (**B**) β-CD presented in the lateral plane.

For the molecular docking simulations, both units of β-CD were used; their front and side views are shown in (Figure 6A,B), respectively. It is noted that both units will contribute to the molecular docking simulations, providing the ES with greater opportunities for interactions with β-CD, and thus showing the actual region where the ligand has the highest affinity for the target molecule/ligand.

**Figure 7:**
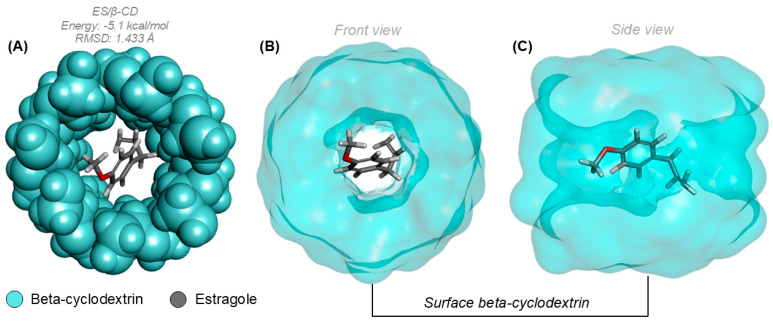
(**A**) Complex formed between β-CD and ES. (**B**) Surface of the complex formed in the frontal plane. (**C**) Surface of the complex with lateral view.

Regarding the prediction calculations made through molecular docking, the analyses showed that a complex was formed between β-CD (cyan) and ES (lead), as observed in Figure 7A. The complex formed between the two molecules displayed a high affinity potential, which is supported by the affinity energy and RMSD values, both of which are −5.1 kcal/mol and 1.433 Å, respectively. This indicates a good affinity index for the ES/β-CD complex. In Figure 7B,C, the surface encompassing the entire β-CD molecule is presented, illustrating the actual position where ES formed the complex.

**Figure 8:**
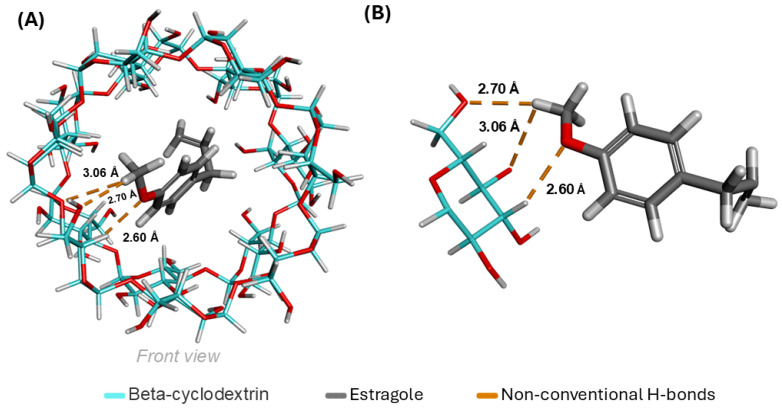
(**A**) Global view of interactions formed from the ES-β-CD complex. (**B**) Specific moieties that formed interactions.

When evaluating the interactions between β-CD and ES, it is possible to achieve (in Figure 8A) a global view of the complex with its interactions, where three unconventional hydrogen bonds (orange) have been established, with distances of 2.60 Å, 2.70 Å, and 3.06 Å. In (Figure 8B), only the parts involved in the formation of the interactions are shown. All contributions are linked through the relationship between carbons and oxygens, with hydrogen acting as an intermediary C-H-O in the three bonds formed by the complex.

### 3.5. MPO-Based Pharmacokinetics Prediction

#### 3.5.1. Topology Analysis and Drug-likeness

**Figure 9:**
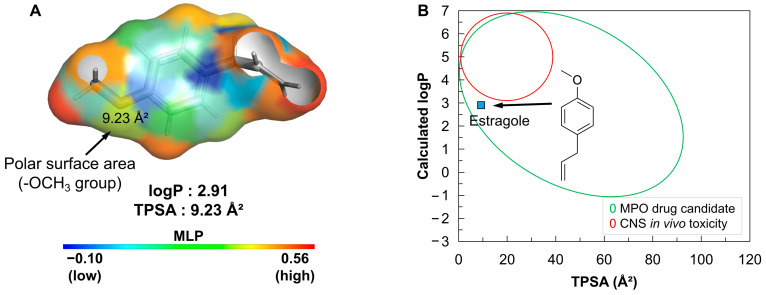
(**A**) Molecular lipophilicity potential (MLP) surface map, where the color spectrum ranges from blue (low MLP) to red (high MLP). (**B**) Alignment between lipophilicity (logP) and polarity (TPSA) for estimating the MPO drug candidate profile and CNS toxicity.

Small compounds based on weak acids that are not very lipophilic (logP ≤ 3) and have a polar surface due to the presence of hydrogen bond acceptor groups (HBA) exhibit overall lipophilicity that allows for good effective cellular permeability (Papp, A→B > 10 × 10^−6^ cm/s), low hepatic clearance (CL int,u < 100 mL/min/kg), and a low incidence of in vivo toxicity. The molecular lipophilicity potential (MLP) allows for the visualization of the polar surface relative to the lipophilic surface, since these attributes are strongly related to the passive diffusion of drug candidates across more selective biological membranes (Figure 9).

#### 3.5.2. Predicted PAMPA Descriptors

**Figure 10:**
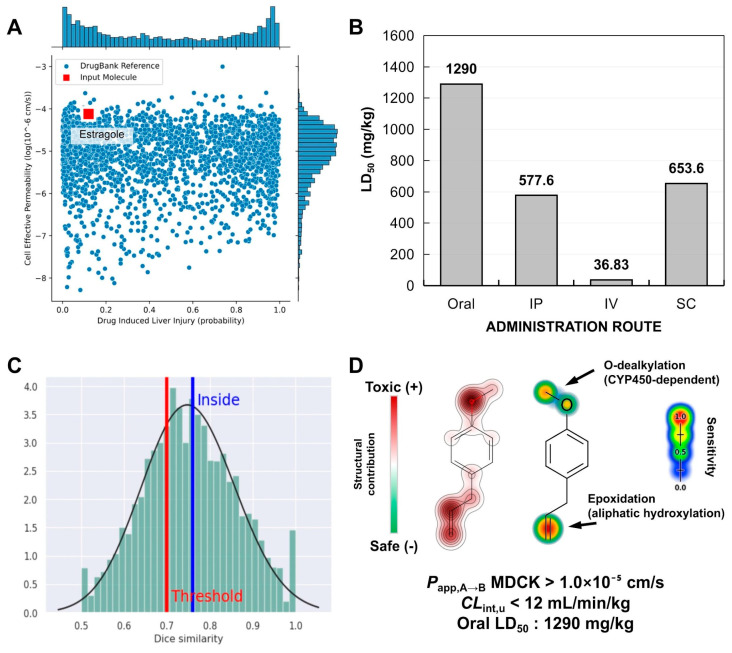
Prediction of pharmacokinetic properties and toxicity expressed in (**A**) a similarity test involving compounds present in the DrugBank^®^ database, (**B**) prediction of lethal dose (LD50) for different routes of administration (oral, IP—intraperitoneal, IV—intravenous and SC—subcutaneous), (**C**) statistical limit of the similarity test of the predictions of (**D**) oral toxicity site and CYP450-dependent metabolism site.

In this study, PAMPA descriptors were predicted to estimate the absorption of estragole in selective cell membranes. The prediction of these descriptors is linked to the selection of the most promising drug candidates with optimized oral absorption. A structural similarity test with compounds from the DrugBank^®^ database, guided by a machine learning model, showed that estragole resides in a physicochemical space formed by compounds with high effective cell permeability and a low likelihood of inducing liver damage through metabolic activation (Figure 10A).

#### 3.5.3. Site of Metabolism and Toxicity Prediction

Predicting metabolism sites is crucial for establishing correlations between liver metabolism and the occurrence of acute toxic responses, as reported in previous studies. Phase I drug metabolism, driven by cytochrome P450 (CYP450) enzymes, involves a series of oxidation and reduction reactions that affect oxygenated groups and unsaturated centers. These reactions lead to the formation of secondary metabolites, which can be both effective and highly reactive. These changes not only influence drug bioavailability but can also affect the daily oral dose administered. One of these metabolites is formed through epoxidation, an unstable intermediate arising from the hydroxylation of unsaturated centers, which can potentially react with macromolecules like proteins and DNA. In this study, an LD50 value of around 1290 mg/kg was predicted for the oral administration route, indicating a class 4 toxicity risk, which suggests that this compound has a low probability of being lethal due to metabolic activation, even though toxic responses are evident. On the other hand, LD50 values < 800 mg/kg for intraperitoneal (IP), intravenous (IV), and subcutaneous (SC) administration routes suggest that the oral route is the most favorable for the therapeutic application of estragole (Figure 10B). The machine learning model used to predict the metabolism site indicated that the compound falls within the statistically reliable threshold of the structural similarity test (similarity > 0.7) with other toxic substructures (Figure 10C). It was possible to observe that the methoxy substructures (-OCH3) and the isolated alkene (R-C=C-R) present in the alkyl side chain, linked to the aromatic substructure of estragole, negatively contribute to the toxicity test, suggesting that they may be reactive or metabolically unstable structures (Figure 10D).

## 4. Discussion

SEM is a powerful technique that provides detailed images of surface morphology, topography, structural and chemical information about various samples. By examining the surface morphology of materials as shape, size, and texture, SEM can reveal microstructural features, associate these characteristics with specific properties and potential defects, and ultimately optimize functional applications [39]. This study acquired SEM images of β-CD and ES/β-CD, and these images were crucial for understanding how the complex formation affects the physical structure of cyclodextrins to highlight the morphological differences between the pure β-cyclodextrin and its estragole-complexed form. The observed changes, such as particle aggregation or smoothness or loss of crystalline structure, serve as evidence of complexation in the β-CD structure, and likely indicate the successful incorporation of estragole into the complexation system (ES/β-CD).

Several terpenes have already demonstrated significant results regarding their antimicrobial and modulating activity [11,40]. According to Soltani et al. (2023), eugenol, a compound from the same class as estragole, interacted with the MexA and AcrA pumps in *P. aeruginosa* and *E. coli*, resulting in a significant reduction in the expression of their genes compared to those not treated with the terpene [41]. The antibiotic resistance of the treated bacteria significantly decreased (*p* < 0.05), indicating that monoterpene can enhance the sensitivity of strains to antibiotics and may help inhibit resistance to these drugs. Furthermore, according to Cheng et al. (2024), eugenol showed antibacterial action when associated with other substances, revealing its synergistic inhibitory potential and action on the trace elements of the treated bacteria [42].

The literature indicates that the monoterpene estragole has demonstrated an antibacterial effect during in vitro [10,11,43] and in vivo assays using rat models and adult zebrafish [11]. For example, studies involving the essential oil of *Croton zehntneri*, which presented estragole as one of its major components, and with the isolated compound, showed potential activity against the Gram-positive bacterial strains evaluated, good antifungal and cytotoxic activity [44]. The same compound was also found in the essential oil of *Ocimum basilicum* L., which has shown an association with strong antibacterial and larvicidal activities [45]. Batista et al. (2023) have reported a promisor antimicrobial potential of modulation of the antibiotic gentamicin in an adult zebrafish model infected with *Staphylococcus aureus* and *Escherichia coli* [11].

The mechanism of bacterial resistance involves a range of adaptive strategies that bacteria use to survive exposure to antibiotics. The efflux system is a critical mechanism of bacterial resistance responsible for transporting antibiotics out of the bacterial cell. The efflux pumps NorA and MepA present in *S. aureus* bacteria are proton pumps that depend on a driving force. NorA, a member of the Major Facilitator Superfamily (MFS), has been extensively studied in light of its role in expelling hydrophilic fluoroquinolones, such as ciprofloxacin, and other antiseptics from the cell [46,47,48]. Similarly, MepA, which belongs to the Multidrug and Toxic Compound Extrusion (MATE) family, contributes to resistance by extruding multiple drugs, though it differs from NorA in that it uses a sodium ion gradient for its function [49,50]. Several compounds have been identified to interact with these pumps, either blocking or modifying their action. *Ethidium bromide* is a substrate that has been widely used in several experiments to search for new efflux pump inhibitors and has been used as a model to prove the efficacy of efflux pumps in bacteria, while CCCP, chlorpromazine, reserpine, and verapamil are known inhibitors of these pumps [48,51,52].

Complexation with cyclodextrins (CDs) improves the pharmacokinetic characteristics of many bioproducts. The most notable property of CDs is their ability to modify the physicochemical characteristics of molecules accommodated within their internal cavity, forming an inclusion complex and increasing the solubility in aqueous solutions [19,53,54]. Complexation can also alter chemical reactivity and reduce oxidation without compromising the substance’s ability to cross biological membranes [21]. According to the results obtained, the MIC of ES/β-CD did not directly affect the strains tested, corroborating findings on the effect of the isolated estragole and beta-cyclodextrin complex against the strains *S. aureus* and *E. coli* [11]. Coêlho et al. (2016) showed that the terpene potentiated the action of the antibiotic norfloxacin when associated with the 1199B strain, corroborating the results of this study. However, in association with ethidium bromide, its MIC was reduced, which does not corroborate our study, in which the MIC was increased for *S. aureus* 1199B [55].

The increase in the minimum inhibitory concentration (MIC) observed in the complex into β-CD may be attributed to the slow kinetic velocity of the transfer of estragole from β-cyclodextrin (β-CD) to a hydrophilic medium at 37 °C, likely due to the stabilization of estragole within the cyclodextrin cavity. These findings align with results from previous studies in the literature [56,57].

The data from the molecular docking simulations show that the ES had good affinity with β-CD, backed by one of the main analysis criteria, which is the position in which the ligand formed the complex. The ES settled in the inner part of the β-CD, showcasing a high degree of specificity with the inner region of the target molecule. Another aspect to consider is the values resulting from complex formation, like the significance of the affinity energy contribution and the variations in root mean square deviation (RMSD), which aim to identify the best conformation during molecular fitting. Among the data, it is noteworthy that the complex showed a strong affinity energy result with a low RMSD variation, indicating the spontaneity of the ES/β-CD complex formation [43].

The data on the interactions established by the ES-β-CD complex indicated that the ES formed three unconventional hydrogen bonds with distances of 2.60 Å, 2.70 Å, and 3.06 Å. This shows that the ES had a good number of (C-H-O) bonds, which directly benefits the target molecule/ligand relationship, as unconventional hydrogen bonds play important roles in chemical and biological structures.

In a topological analysis of the MLP (Figure 7A), it can be seen that the isolated alkene in the aliphatic side chain of estragole contributes to the formation of a surface with a strong lipophilic potential (orange to red color spectra). On the other hand, the methoxy group (R-OCH_3_) forms an HBA surface with a topological polarity of about 9.23 Å^2^, creating a slightly water-soluble molecular surface near the aromatic structure (yellow to blue color spectra), resulting in a logP of about 2.91. Despite the compound’s low polarity, estragole sits in a physicochemical space with a low incidence of in vivo toxicity due to its low overall lipophilicity (logP ≤ 3), resulting in an MPO score of 4.54 (Table 1) that indicates the compound is an excellent MPO drug candidate (Figure 7B), essentially aligning with Pfizer, Inc.’s drug-likeness trends for selecting safer compounds with good pharmacokinetic viability.

This initial prediction supports the Papp, A→B, estimated at around 10^−5^ cm/s in the MDCK cell line, indicating that the compound has high permeability in more selective cellular membranes (Table 2), such as the blood–brain barrier (BBB). On the other hand, the discrepancy observed in the predicted Papp, A→B in the Caco-2 cell model, with values ranging from 5.8 × 10^−6^ to 5.2 × 10^−5^ cm/s, reveals an ambiguity in the predicted cellular permeability for less selective environments (Table 2). When paired with the low likelihood of the compound undergoing passive extracellular efflux by P-gp and the low clearance rate, with predicted CL values of <12 mL/min/kg, a high likelihood of the compound exhibiting viable oral bioavailability for achieving the therapeutic effect is estimated. Additionally, it was observed that the lipophilicity of estragole is directly related to its systemic distribution after intestinal absorption. The predicted volume of distribution (VD) of >1.6 L/kg, based on the consensus predictive test, indicates that the bioavailable molecular fraction (found freely in the blood) of the compound is widely distributed in biological tissues, including cellular membranes, due to its moderate lipophilicity (Table 2).

The-OCH_3_ group constitutes a CYP450 2D6-dependent O-dealkylation site (Table 2), indicating that the metabolic processes involving the biotransformation of estragole promote the formation of hydroxylated secondary metabolites. On the other hand, the isolated alkene shows high structural specificity for epoxidation sites (Figure 10), stemming from aliphatic hydroxylation, which supports the risk of the compound inducing a mutagenic response (Table 2). This consensus analysis suggests that estragole has a reactive center based on epoxidation, which is capable of inducing an organic toxic response. However, the predicted oral LD50 highlights the compound’s low likelihood of being lethal when administered through intestinal absorption and metabolic activation.

## 5. Conclusions

Previous studies have highlighted the potential antibacterial action of estragole and its ability to modulate bacterial resistance by interfering with the efflux pump. In this new study, we developed a complex with cyclodextrin. The molecular docking results indicate that the interaction between estragole (ES) and β-cyclodextrin (β-CD) may promote favorable system formation. This is supported by evaluation parameters such as interaction energy, changes in root mean square deviation (RMSD), and the formation of three unconventional hydrogen bonds. The molecular docking simulation data suggest a strong affinity for the formation of the ES/β-CD complex. Unlike isolated estragole, the ES/β-CD complex did not demonstrate direct interference with the efflux pump; however, it did influence the action of norfloxacin against the bacterial strain *S. aureus* 1199B, likely by affecting membrane stability and facilitating the transport of the antibiotic into the cell. Pharmacokinetic predictions made via molecular predictive optimization (MPO) indicate that the compound has moderate lipophilicity, which facilitates its distribution in biological tissues. Its high cellular permeability and low organic toxicity risk were also noted. These results indicate its possible and relevant association as an adjuvant in antibiotic therapy to reduce multidrug-resistant bacteria; however, new in vivo assays are necessary to confirm this effect.

## Figures and Tables

**Table 1:** Physicochemical properties of estragole calculated and applied to the scoring and drug-likeness evaluation criteria of the MPO system from Pfizer, Inc.

Property	Value	T⁰
logP	2.91	1.00
logD at pH 7.4	2.91	0.54
MW	148.21 g/mol	1.00
TPSA	9.23 Å^2^	0.00
HBD	0	1.00
pKa	−4.82	1.00
MPO score	4.54	
Pfizer rule	Accepted	

**Table 2:** Predicted pharmacokinetic attributes of estragole, expressed in descriptors of PAMPA, metabolism and organic toxicity.

Property	ADMETlab 3.0	Deep-PK	PreADMET
*P*_app,A→B_ Caco-2	3.04 × 10^−5^ cm/s	5.20 × 10^−5^ cm/s	5.80 × 10^−6^ cm/s
*P*_app,A→B_ MDCK	2.31 × 10^−5^ cm/s	2.23 × 10^−4^ cm/s	1.72 × 10^−5^ cm/s
*CL*_int,u_	11.721 mL/min/kg	6.78 mL/min/kg	-
P-gp efflux	Non-substrate	Non-substrate	Non-inhibitor
VD	2.35 L/kg	1.62 L/kg	-
CYP2C9 substrate	Substrate	Non	Non
CYP2D6 substrate	Substrate	Substrate	Weakly
CYP3A4 substrate	Non	Non	Non
Ames Mutagenicity	0.52	Safe	Yes
DILI—Liver injury	0.38	Safe	-

## Data Availability

Data are contained within the article.
